# 672 Nurse First Interdisciplinary Round on the Burn Unit

**DOI:** 10.1093/jbcr/iraf019.301

**Published:** 2025-04-01

**Authors:** Shawna Thomas, Caitlin Sayers, Natalie Fitzgerald, Audrey O’Neil, Brett Hartman

**Affiliations:** Eskenazi Health; Eskenazi Health; Richard M. Fairbanks Burn Center; Richard M. Fairbanks Burn Center; Eskenazi Health

## Abstract

**Introduction:**

The Registered Nurse (RN) holds an integral role in providing patient care and leading outcomes for patients during their hospitalization. Historically, during Interdisciplinary Rounds (IDR) the physician team leads the discussion, while nursing is represented by nursing leadership, as opposed to the bedside RN. Nurse First IDR is a quality improvement project that was implemented on this Adult Verified Burn Unit in December of 2022, which allows the bedside RN to lead IDR discussions. This project was implemented to improve RN engagement ensuring their work is valued, decrease RN turnover, and improve patient infection rates such as CAUTI and CLABSIs.

**Methods:**

Prior to implementation of Nurse First, the Unit Council on the burn unit developed a rounding sheet to guide IDRs. At implementation, the assigned bedside RN leads the IDR discussion by providing pertinent information including post-burn day, post-operation day, hospital day, fall risk, current pain score, calorie intake, wound overview, dressings, and dressing change tolerance. The RN also reviews all current inserted devices, leading the discussion of necessity and opportunity for removal. Lastly, any significant events that have occurred in the last 24 hours is brought forward for discussion with the interdisciplinary team. Following the RN, the physician team provides updates to the team to continue IDR discussions. Employee engagement, nurse turnover, and infection rates were collected following implementation and compared to pre-implementation outcomes.

**Results:**

The 2022 employee engagement survey results for the burn team demonstrated an overall improved engagement with two specific questions. “My work environment inspires me to perform at my very best” improved from 69% in 2019 to 86% in 2022. “I will definitely not be looking for a position elsewhere within the next year” improved from 60% to 83% from 2019 to 2022. Burn nurse turnover continues to decrease post-implementation of Nurse First, improving from 18% in 2023 to 10% year-to-date, with first-year turnover at 3%. Infection rates for CAUTI also improved from a 2022 pre-implementation Standardized Infection Ratio (SIR) of 0.322 to a post-implementation ratio of 0.000 in 2023. The CLABSI SIR increased slightly from 2022 to 2023 from 0.000 to 0.642 representing two CLABSIs that occurred on a medicine patient, not admitted to the burn service.

**Conclusions:**

Implementation of Nurse First improved employee engagement and positively impacted nurse turnover in the burn unit. Infection rates for CAUTI decreased while there was a slight uptick in CLABSI related to a medicine patient. Nurse First engages RNs in their work and increases the perception of feeling valued.

**Applicability of Research to Practice:**

Applicability to Research: Nurses are integral in a patient’s healing and outcomes and should be included in Interdisciplinary Rounds providing patient care information.

**Funding for the Study:**

N/A

